# Molecular mechanisms of flavonoid accumulation in germinating common bean (*Phaseolus vulgaris*) under salt stress

**DOI:** 10.3389/fnut.2022.928805

**Published:** 2022-08-29

**Authors:** Qi Zhang, Guangyue Zheng, Qi Wang, Jixing Zhu, Zhiheng Zhou, Wenshuo Zhou, Junjie Xu, Haoyue Sun, Jingwen Zhong, Yanhua Gu, Zhengong Yin, Yan-li Du, Ji-dao Du

**Affiliations:** ^1^Legume Crop Laboratory, Agricultural College, Heilongjiang Bayi Agricultural University, Daqing, China; ^2^Qiqihar Branch of Heilongjiang Academy of Agricultural Sciences, Qiqihar, China; ^3^Crop Resources Institute of Heilongjiang Academy of Agricultural Sciences, Harbin, China; ^4^National Cereals Technology Engineering Research Center, Daqing, China

**Keywords:** flavonoids (rutin) content, common bean, germinating, salt stress, mechanism, phenylpropanoid biosynthesis, flavonoid biosynthesis

## Abstract

Flavonoids are important secondary metabolites, active biomolecules in germinating beans, and have prominent applications in food and medicine due to their antioxidant effects. Rutin is a plant flavonoid with a wide biological activity range. In this study, flavonoid (rutin) accumulation and its related molecular mechanisms in germinating common bean (*Phaseolus vulgaris*) were observed at different time points (0–120 h) under salt stress (NaCl). The rutin content increased from germination onset until 96 h, after which a reducing trend was observed. Metabolome analysis showed that salt stress alters flavonoid content by regulating phenylpropanoid (ko00940) and flavonoid (ko00941) biosynthesis pathways, as well as their enzyme activities, including cinnamyl-alcohol dehydrogenase (CAD), peroxidase (POD), chalcone isomerase (CHI), and flavonol synthase (FLS). The RNA-seq and quantitative real-time PCR (qRT-PCR) analyses also showed that these two pathways were linked to changes in flavonoid content following salt treatment. These results reveal that salt stress effectively enhanced rutin content accumulation in germinating beans, hence it could be employed to enhance the functional quality of germinating common beans.

## Introduction

The common bean (*Phaseolus vulgaris* L.) is an annual legume crop that has been cultivated in temperate and semitropical regions for ~8,000 years (1). Common beans constitute an essential part of the human diet not only because of their protein-rich seed but also because they contain active phytochemicals, which are beneficial for health (2), including proteases, galacto-oligosaccharides, phytic acid, and flavonoids (3).

Flavonoids are an abundant and biologically active family of natural plant products that offer a range of health benefits (4). For instance, several flavonoids have anticancer functions, both *in vitro* and *in vivo* (5, 6). Some of the reported mechanisms by which flavonoids exert their anticancer functions include (1) preventing new cancer cell development, (2) restraining carcinogens that turn into activation sites, and (3) preventing compound metabolism and reducing their toxicity (7). Recently, with the ever-growing food quality concerns, the international market has shown a growing interest in flavonoid production, with high demand and a growing compound rate of 16.5%.

Plants are the most common material used for flavonoid extraction (7). These flavonoids have a C6–C3–C6 carbon skeleton with two phenyl aromatic rings, along with a heterocyclic ring (8). Flavonoids can be split into several subclasses according to basic skeleton substitution and B-ring attachment, such as chalcones, stilbenes, aurones, flavanones, flavones, isoflavones, phlobaphenes, dihydroflavonols, flavonols, leucoanthocyanidins, proanthocyanidins, and anthocyanins (9, 10). Rutin (C_27_H_30_O_16_) is a relatively large and representative flavonoid in leguminous crops (11–13), which could also be used as a representative flavonoid for studying plant nutrition (14). In plants, certain flavonoids, including rutin, have been proven to play an important role in plant growth, development, hormone signaling, facilitating pollen–tube germination, plant–microorganism interactions, and biotic and abiotic stresses (15, 16).

Legumes are potential raw materials used for producing flavonoids for nutritional and dietary applications (4). However, abiotic stress (e.g., drought, salt, and cold) can affect the flavonoid biosynthetic pathway by regulating flavonoid transport and the expression and accumulation of related proteins, finally affecting the flavonoid content in plants (17). Previous studies have shown that salt stress significantly inhibits the germination of legume crops (including *Lathyrus sativus, Vicia sativa, Vigna radiata*, and *Vigna unguiculata*) but increases the flavonoid content in their seeds (18). Besides, another study reported significant differences in the flavonoid content and anti-inflammatory effects of soybean when seeds were germinated under different abiotic stress treatments (19). It has also been reported that abiotic stress could increase the content of total phenolic compounds and total flavonoids in soybean (*Glycine max*) and mung bean (*V. radiata*) sprouts (19, 20). Therefore, the regulation of flavonoid biosynthesis is considered an important regulatory mechanism of legume crops in response to salt stress (18). In another study, Gu et al. (21) demonstrated that moderate drought stress treatment could increase flavonoid content in plants. From a nutritional viewpoint, it could be concluded that exposing plants to appropriate stress treatment is an effective strategy for increasing the flavonoid content in food. However, few studies have reported the correlation between germinating common beans and flavonoids. This study determined the accumulation of rutin (used as the representative flavonoid in the common bean) in different common bean tissues at different germination stages under salt stress. Furthermore, the effect and mechanism of salt stress on total flavonoid accumulation in common bean sprouts were evaluated through metabolome and transcriptome analysis. The results presented herein provide a theoretical basis and new insights for improving the flavonoid content of common bean sprouts.

## Materials and methods

### Common bean materials and treatments

A local common bean cultivar (Longjiang Black Yun) was used as testing material, and its seeds were provided by the National Coarse Cereals Technology Engineering Research Center (Heilongjiang, China). The seeds were surface-sterilized with sodium hypochlorite (NaClO) and washed three times with distilled water. Germination was done in a germination box (BSC-250, Boxun, Shanghai, China) at a constant temperature of 25°C without light. Salt stress was simulated using 70 mmol/L NaCl (22) and was added at different times, as illustrated in Supplementary Table 1. The seeds were exposed to salt stress for 0, 24, 48, 72, 96, and 120 h for sprouting, and the germinating common beans were sampled to measure the rutin content. Different tissues with the highest flavonoid content in the germinating common beans were isolated since they were most suitable for further research. Flavonoid extraction was done using the method described by Liu et al. (23), while the rutin content was determined using HPLC (AB SCIEX, shimadzuLc-20A, MA, USA) from the Customs Quality Inspection Center (Dalian, Liaoning, China), using rutin (C_27_H_30_O_16_) (SR8250, Solarbio, Beijing, China) as the reference (24–26). The standard rutin curve is shown in Supplementary Figure 1.

### Metabolome and transcriptome analysis

Bean sprouts, without cotyledons, in the 3d+S treatment were taken at three time points (3d+S-0h, 3d+S-12h, and 3d+S-24h) and used as test samples for metabolome and transcriptome analysis, with three biological replicates at each time point. The hours (0, 12, and 24 h) represent the time salt stress was introduced at 3 days. The broadly targeted metabolome was determined using the UPLC-ESI-MS/MS system (Nexera X2, SHIMADZU, Japan) and ESI-Q TRAP-MS/MS (4500 QTRAP, Applied Biosystems, USA) from Matwell Biotechnology Company (Wuhan, China) (Supplementary Figure 2). The HPLC conditions included liquid-phase chromatographic column (Waters ACQUITY UPLC HSS T3 C18 1.8 μm, 2.1 mm ^*^ 100 mm), mobile phase (phase A was ultrapure water with 0.04% acetic acid while phase B was acetonitrile with 0.04% acetic acid), flow rate (0.35 ml/min), column temperature (40°C), and injection volume (4 μl). The mass spectrometry conditions included an electrospray ionization temperature of 550°C, a mass spectrometer voltage of 5,500 V, and a curtain gas pressure of 30 psi. Each ion pair in a triple quadrupole was scanned according to the optimized declustering potential and collision energy (27). The reagents (including methanol, acetonitrile, and ethanol) were bought from Merck (Darmstadt, Germany), while the standards were bought from Sigma (Sigma-Aldrich, Shanghai, China). The blank reagent sample was run during the detection process in order to clean the detection residue. The qualitative analysis of metabolites in samples was based on the MetWare database, so the isotopic and repeating signals (including K^+^, Na^+^, and NH${}_{4}^{+}$) were removed. The metabolite identification method was based on a self-built database, including standard products and experimental samples. The secondary mass spectrometry data were collected based on the mass spectrometry MIM-EPI mode, and the unique Q1 (molecular ion), Q3 (characteristic fragment ion), RT (chromatographic retention time), DP (declustering potential), and CE (collision energy) of each metabolite were constructed by combining with manual analysis of the spectrum. Subsequently, the experimental samples were quantitatively detected using the multiple reaction monitoring (MRM) mode, which could eliminate nontarget ion interference and increase the accuracy and reproducibility of the quantification (27). After screening, the Analyst 1.6.3 software was used to process the mass spectrometry data, and the MultiQuant software to open the sample off-machine mass spectrometry file to integrate and calibrate the chromatographic peaks. The peak area of each chromatographic peak represents the relative content of the corresponding substance. The quality control (QC) in this study was used with mixed samples and internal standards (L-2-chlorophenylalanine, CAS: 103616-89-3) using the processing method applied for the samples. During the instrumental analysis, a QC sample was inserted into every 10 detection and analysis samples to monitor the repeatability of the analysis process. Fold change (FC) and VIP value analysis were used to find differentially altered metabolites (DAMs), and the screening conditions for DAMs were VIP ≥ 1 and FC ≥ 2 or FC ≤ 0.5 (28). The Kyoto Encyclopedia of Genes and Genomes (KEGG) database was used to annotate the information from the DAMs (29), with the *p*-value set to <0.05.

Also, the bean sprouts without cotyledons at three time points (3d+S-0h, 3d+S-12h, and 3d+S-24h) were used as transcriptome samples with three biological replicates at each time point. The transcriptome of samples was determined using HiSeq X Ten (Illumina, CA, USA) from the Biomarker Company (Beijing, China), and the transcriptome data were analyzed on the BMKCloud platform. The TopHat2 software (30) was used for sequence alignment of clean reads with a reference genome, while fragments per kilobase of transcript per million fragments mapped was used to measure the transcript or gene expression levels (31). The FC and false discovery rate (FDR) were calculated to determine the differentially expressed genes (DEGs). The value of FC was the average value in each group, which was calculated by merging three biological replicates in one treatment. The screening conditions for DEGs were FC ≥ 2 and FDR < 0.01 (28). The KEGG database was used to annotate the information from the DEGs (29), with the *p*-value set to < 0.05.

### Physiology and qRT-PCR analysis

Enzyme activities, including cinnamyl-alcohol dehydrogenase (CAD) (32), peroxidase (POD) (33), chalcone isomerase (CHI) (34), and flavonol synthase (FLS) (35), in the enriched pathways were determined with an ELISA Kit (Michy, Suzhou, China), and data were acquired using a microplate reader (SpectraMax^®^ 190, Molecular Devices, CA, USA). The data were then analyzed using ANOVA at a significance level of *p* < 0.05 on the SPSS19.0 platform.

The RNA of each sample was extracted with the *Trelief*^TM^ RNA Pure Plant Kit (TSP412, Tsingke, China), and a NanoDrop (OneC, Thermo, USA) was used to detect quality and concentration. Quality RNA was reverse-transcribed into cDNA with the *Evo M-MLV* RT Premix (Accurate-Biotechnology, AG11706, Hunan, China). Primers of DEGs enriched in CAD (*Phvul.002G144800* and *Phvul.003G029500*), POD (*Phvul.001G143300, Phvul.007G008400* and *Phvul.008G249900*), and FLS (*Phvul.003G216600*) were designed using Primer Premier 5.0, while *Pvactin-11* was selected as the reference gene; the primer sequences are shown in Supplementary Table 2. The qRT-PCR program was conducted on a Light Cycler 480II (Roche Diagnostics, Switzerland), using a Hieff UNICON^®^ Universal qPCR SYBR Green Master Mix (Yeason, 11184ES03, Shanghai, China). Subsequently, the relative gene expression level was computed using the 2^−ΔCt^ method (36).

## Results

### The rutin content in germinating common bean

**Figure 1:**
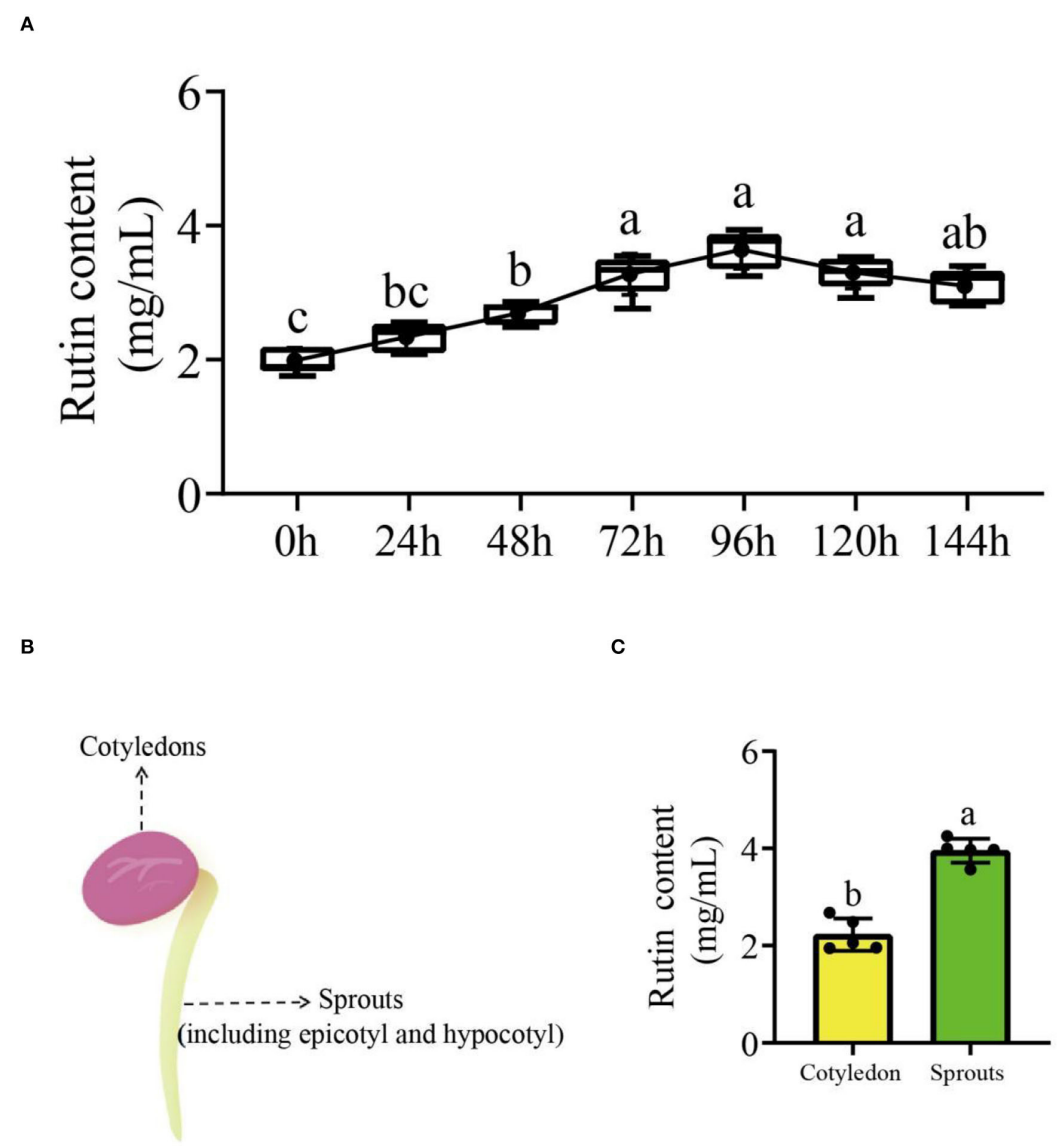
The rutin accumulation trend in germinating common beans and tissue analyses. **(A)** The variation curve of rutin accumulation at different germination times; **(B)** schematic illustration of different tissues that were sampled; **(C)** the rutin content in different tissues of germinating common beans. Lowercase letter(s) indicate significant differences (α = 0.05). Each black point represents the average value of replicates.

The rutin content of common beans without germination was significantly lower (1.99 mg/ml; *p* < 0.05) at different germination times compared to common beans with germination. The rutin content of common bean sprouts at different germinating times increased with time and peaked at 96 h, after which a reducing trend was observed. Compared with other germination times, the periods from 72 to 120 h were more suitable for germinating bean sprouts due to the rutin content, while 96 h could be considered optimal (Figure 1A). Bean sprouts at 96 h were divided into two parts for the determination of flavonoid content (Figure 1B); cotyledons and sprouts (including epicotyl and hypocotyl). Sprouts had a significantly (*p* < 0.05) higher rutin content than cotyledons (Figure 1C). The rutin content in different tissues revealed that sprouts are a suitable target tissue for further research.

### Effects of salt stress on rutin content

**Figure 2:**
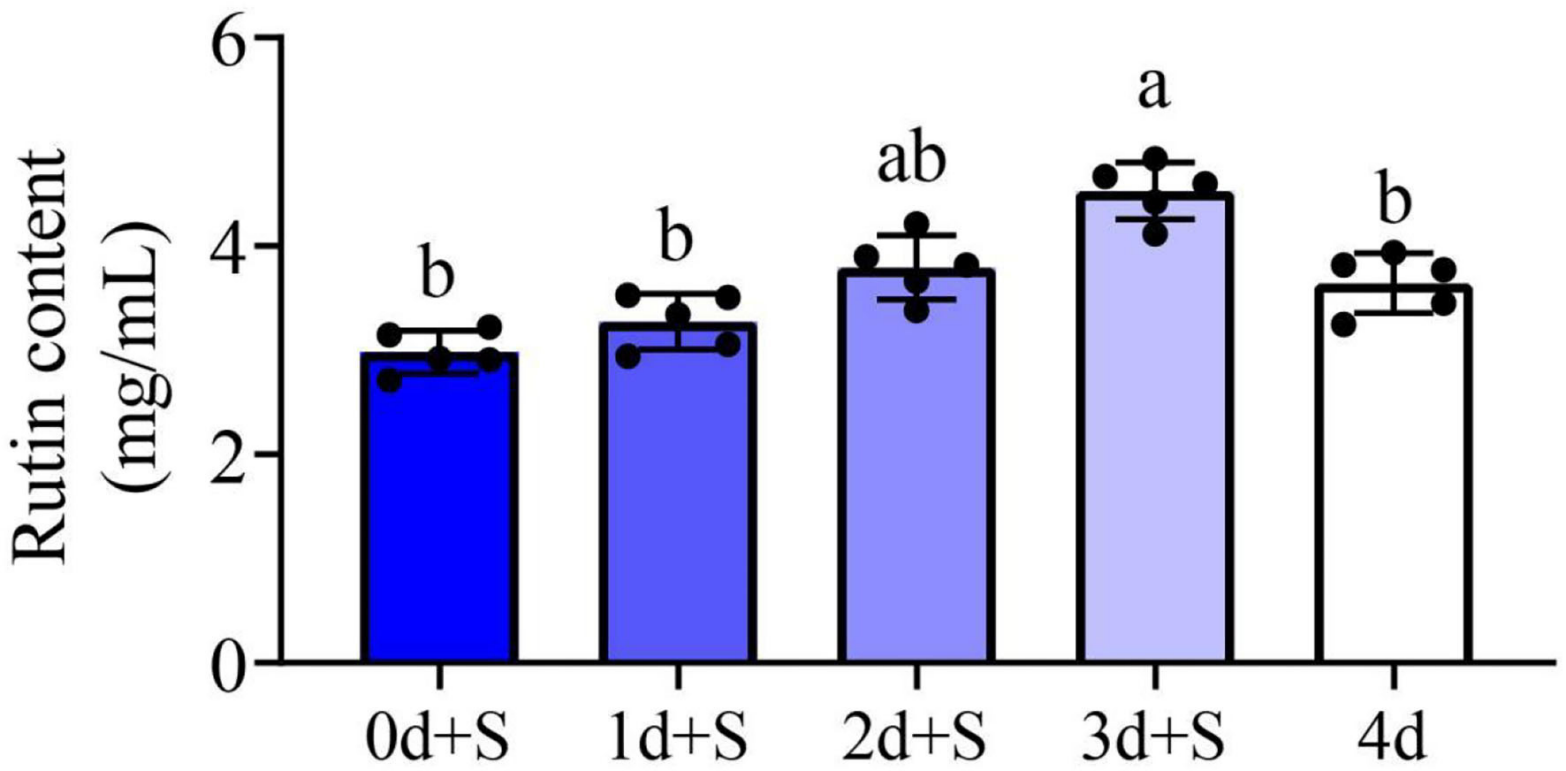
The rutin content in different treatments. Different columns represent different treatments, and dark to light color represents the salt addition time from long to short. Lowercase letter(s) indicate significant differences (α = 0.05). Each black point represents the average value of replicates.

The rutin contents under different salt exposure times were determined in this study. The result revealed differences in rutin content accumulation at different periods of salt addition. Compared to the treatment without salt (4 days), the rutin content of 2d+S and 3d+S treatments increased, while that of treatments 0d+S and 1d+S decreased (Figure 2). Short-term salt stress, thus, accelerated rutin content accumulation more than long-term stress, and the 3d+S treatment could be used as an optimal treatment to study the mechanism of rutin content accumulation.

### Metabolome and transcriptome analysis

**Figure 3:**
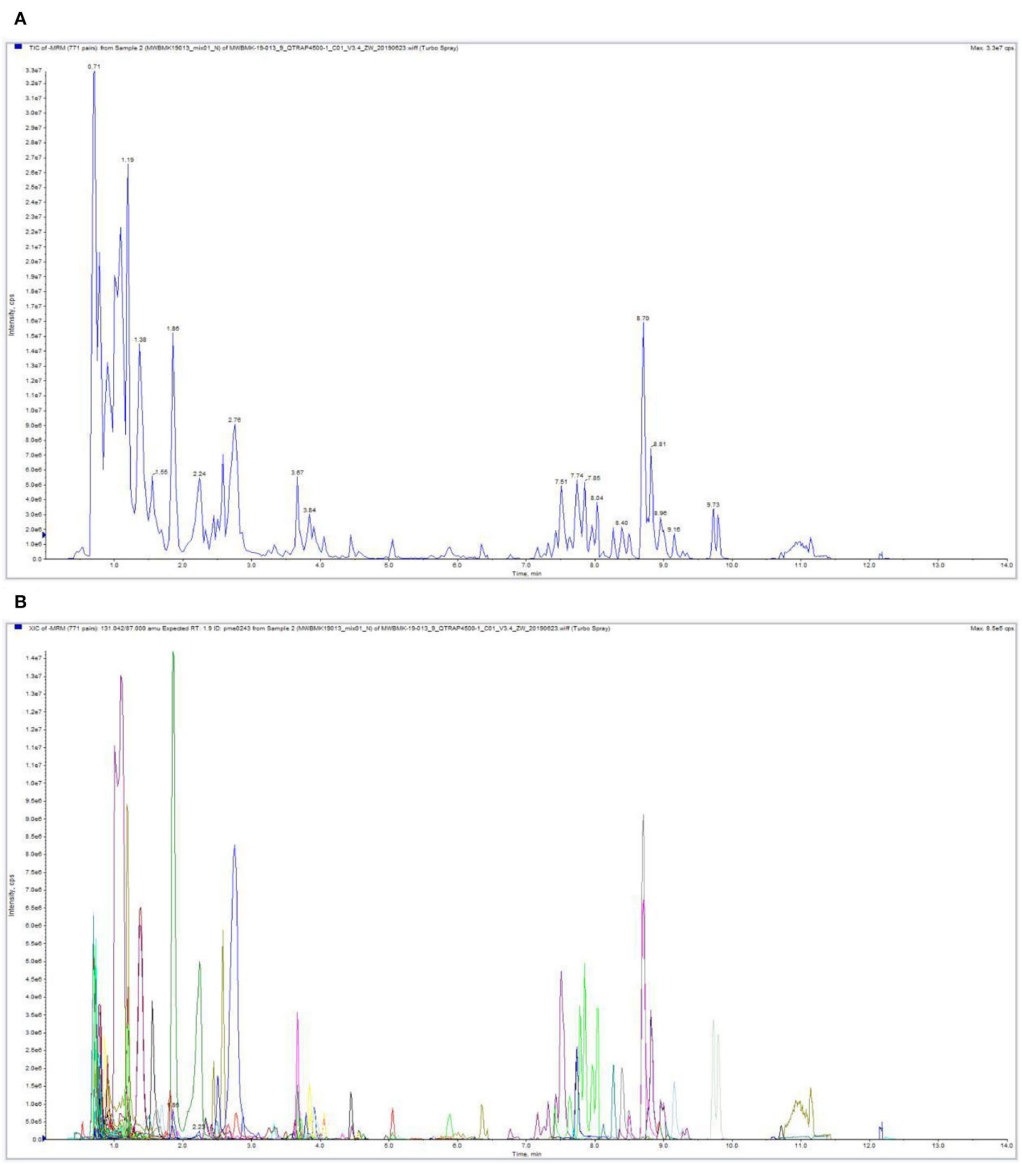
The overlay analysis of the QC-TIC and multipeak detection in MRM analysis of the metabolome. **(A)** The ion current of one sample, as revealed by mass spectrometry detection. **(B)** A multipeak detection plot of the metabolites in the multiple reaction monitoring mode.

The study added a time point (12 h) to enhance broad target metabolome accuracy, with three time points (3d+S-0h, 3d+S-12h, and 3d+S-24h) in the 3d+S treatment, and each time point had three biological replicates. The raw data were uploaded to the Zenodo database (doi.org/10.5281/zenodo.6820497). A total of 732 metabolites were detected; the detailed information, including Q1, Q3, Rt, molecular weight, and ionization model, is shown in Supplementary Table 3. The overlay analysis of the QC-TIC diagram and the sample multipeak detection of MRM analysis diagram (Figure 3) showed that the data had good reproducibility and reliability, hence they could be used for further analysis.

**Figure 4:**
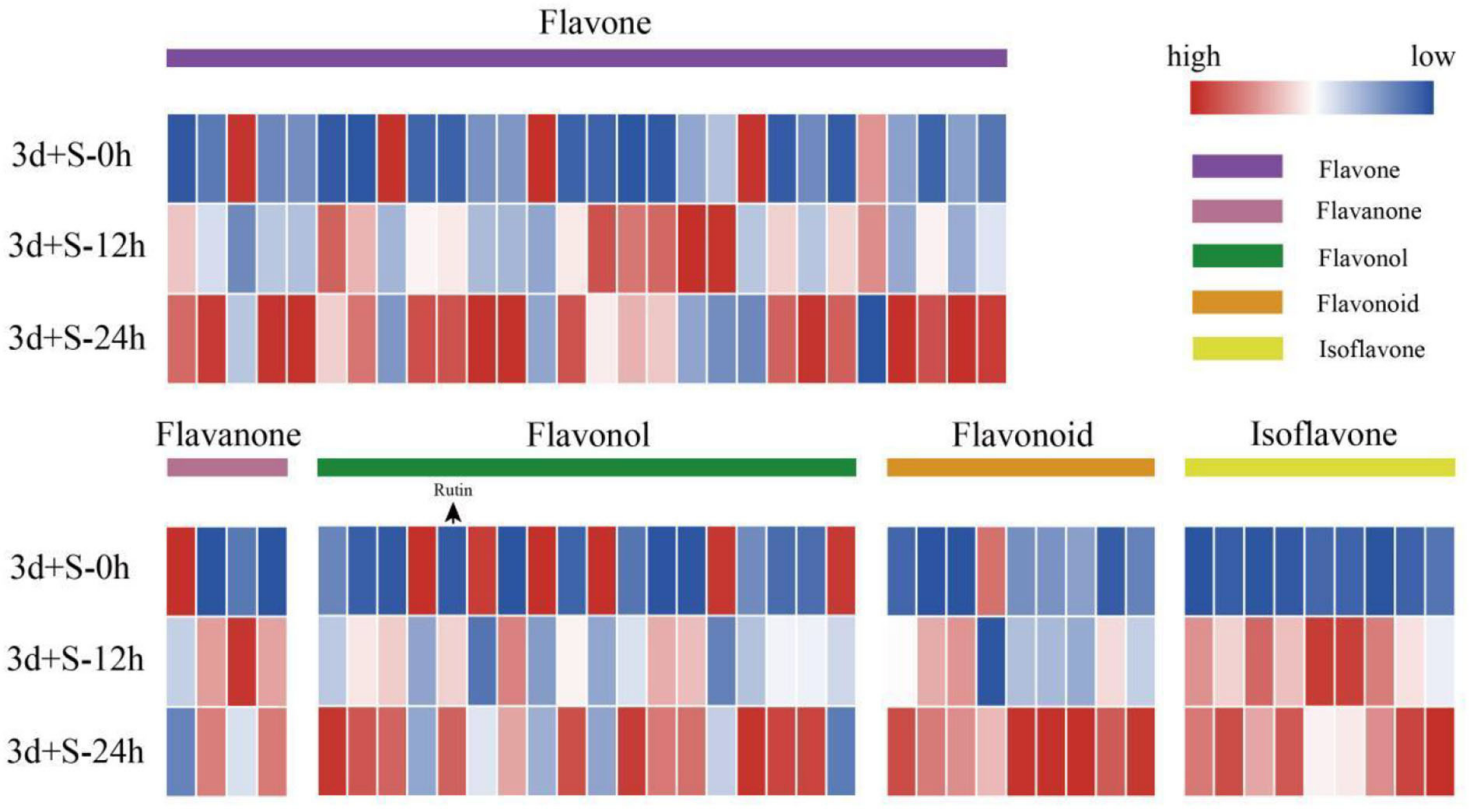
The heatmap of differentially altered metabolites associated with flavonoids. Different colored modules on the heatmap represent different types of flavonoids, while the order from top to bottom is 3d+S-0h, 3d+S-12h, and 3d+S-24h. Blue to red color represents expression from low to high.

In the comparison of 3d+S-0h vs. 3d+S-24h, 86 DAMs were increased while 55 were decreased (Supplementary Table 4). In the comparison of 3d+S-0h vs. 3d+S-12h, 62 DAMs were increased, while 63 were decreased (Supplementary Table 5 and Table 1). In this study, the DAMs in 3d+S-0h vs. 3d+S-24h and 3d+S-0h vs. 3d+S-12h had been combined together for analysis. A total of 189 DAMs were obtained after removing 77 duplicate DAMs, while 68 DAMs were associated with flavonoids after removing 36 duplicate flavonoid DAMs. In these, 68 DAMs were associated with flavonoids, five types of flavonoids (i.e., flavone, flavonol, flavonoid, flavanone, and isoflavone) had different expression levels (Figure 4), and each compound tested by HPLC-MS is listed in Supplementary Table 6. Rutin made up one DAM metabolome, and its content increased significantly. The KEGG enrichment was analyzed for two comparisons. In 3d+S-0h vs. 3d+S-12h, eight pathways had a *p*-value < 0.05 and could thus be used as enrichment pathways (Table 2). In 3d+S-0h vs. 3d+S-24h, five pathways had a *p*-value < 0.05 and could thus be used as enrichment pathways (Table 3). Four candidate pathways were found in the metabolomics analysis, namely, phenylpropanoid biosynthesis (ko00940), flavonoid biosynthesis (ko00941), flavone and flavonol biosynthesis (ko00944), and biosynthesis of secondary metabolites (ko01110).

**Table 1:** The number of differentially altered metabolites (DAMs).

**Groups**	**Total DAMs**	**Up DAMs**	**Down DAMs**
3d+S-0h *vs*. 3d+S-12h	141	86	55
3d+S-0h *vs*. 3d+S-24h	125	62	63

**Table 2:** The enriched pathways of differentially altered metabolites (DAMs) compared 3d+S-0h vs. 3d+S-12h in KEGG analysis.

**No**.	**Ko ID**	**KEGG pathway**	**P-value**	**DAMs**
1	Ko00944	Flavone and flavonol biosynthesis	0.000	pmb0605; pme0199; pme0197; pme3211; pme1622; pme0089
2	Ko00941	Flavonoid biosynthesis	0.001	pme0199; pmf0108; pme0089; pme1521; pme3461; pme1201
3	Ko00945	Stilbenoid, diarylheptanoid and gingerol biosynthesis	0.015	pme1458
4	Ko00940	Phenylpropanoid biosynthesis	0.018	pme3125; pme0030; pma0149; pme3305
5	Ko01110	Biosynthesis of secondary metabolites	0.021	pme0199; pme3261; pme0030; pme3125; pme2555; pmf0558; pme1622; pme3266; pme0089; pme1521; pme1692; pme1201; pme0197; pmf0248; pme3211; pme1002; pme1458; pme3233; pme0026; pmb0449; pme3305
6	Ko00965	Betalain biosynthesis	0.027	pme0030
7	Ko00230	Purine metabolism	0.027	pme1119; pme0193; pme1692; pmb0514; pmb2684; pme2555; pmd0023
8	Ko00910	Nitrogen metabolism	0.033	pme0193

**Table 3:** The enriched pathways of differentially altered metabolites (DAMs) compared 3d+S-0h vs. 3d+S-24h in KEGG analysis.

**No**.	**Ko ID**	**KEGG pathway**	**P-value**	**DAMs**
1	Ko00944	Flavone and flavonol biosynthesis	0.000	pme0374; pme1622; pme3211; pme0197
2	Ko00941	Flavonoid biosynthesis	0.000	pme1201; pmf0345; pme3509; pme1521; pmf0108
3	Ko01110	Biosynthesis of secondary metabolites	0.001	pme1695; pme2801; pme2993; pme0006; pmf0558; pme0303; pme3266; pme3125; pme2122; pme3305; pme0197; pme1622; pme0355; pmf0117; pme2155; pme1201; pmf0345; pme3252; pme1841; pme3233; pme1521; pme3211; pme3261; pme0013; pme3553
4	Ko00940	Phenylpropanoid biosynthesis	0.003	pme0303; pmb0242; pme2993; pma0149; pme3125; pme1695; pme3305
5	Ko00943	Isoflavonoid biosynthesis	0.028	pme3233; pme3509; pme3208; pme3252; pme0355; pmf0117; pme3266; pme3261

The samples of three time points, i.e., 3d+S-0h, 3d+S-12h, and 3d+S-24h in the 3d+S treatment, were used for RNA-seq. The data quality was sufficient for follow-up analysis (Supplementary Table 7) and data were uploaded to the NCBI database (PRJNA746732) (Supplementary Table 8). In the comparisons of 3d+S-0h vs. 3d+S-12h, and 3d+S-0h vs. 3d+S-24h, a total of 1,230 and 1,450 DEGs were found, of which 812 and 904 DEGs were upregulated, respectively, and 418 and 546 were downregulated, respectively (Table 4). All DEGs were analyzed in KEGG enrichment, respectively. A total of 16 pathways were enriched in 3d+S-0h vs. 3d+S-12h (Table 5, while 13 pathways were enriched in 3d+S-0h vs. 3d+S-24h (Table 6). In the transcriptome analysis of 3d+S-0h vs. 3d+S-12h, and 3d+S-0h vs. 3d+S-24h, seven candidate pathways were found. From the combined metabolome and transcriptome results of enriched KEGG pathways, phenylpropanoid biosynthesis (ko00940) and flavonoid biosynthesis (ko00941) responded to salt stress by accumulating flavonoids.

**Table 4:** The number of differentially expressed genes (DEGs).

**Groups**	**Total DEGs**	**Up DEGs**	**Down DEGs**
3d+S-0h *vs* 3d+S-12h	1230	812	418
3d+S-0h *vs* 3d+S-24h	1450	904	546

**Table 5:** The enriched pathways of differentially expressed genes (DEGs) compared 3d+S-0h vs. 3d+S-12h in KEGG analysis.

**No**.	**Ko ID**	**KEGG pathway**	***P*-value**
1	Ko00195	Photosynthesis	0.000
2	Ko00196	Photosynthesis—antenna proteins	0.000
3	Ko00904	Diterpenoid biosynthesis	0.000
4	Ko00360	Phenylalanine metabolism	0.000
5	Ko00940	Phenylpropanoid biosynthesis	0.000
6	Ko00710	Carbon fixation in photosynthetic organisms	0.001
7	Ko00730	Thiamine metabolism	0.008
8	Ko00330	Arginine and proline metabolism	0.009
9	Ko00860	Porphyrin and chlorophyll metabolism	0.012
10	Ko00380	Tryptophan metabolism	0.015
11	Ko00290	Valine, leucine and isoleucine biosynthesis	0.016
12	Ko00908	Zeatin biosynthesis	0.022
13	Ko00906	Carotenoid biosynthesis	0.026
14	Ko00280	Valine, leucine and isoleucine degradation	0.030
15	Ko01220	Degradation of aromatic compounds	0.033
16	Ko00591	Linoleic acid metabolism	0.047

**Table 6:** The enriched pathways of differentially expressed genes (DEGs) compared 3d+S-0h vs. 3d+S-24h in KEGG analysis.

**No**.	**Ko ID**	**KEGG pathway**	***P*-value**
1	Ko00195	Photosynthesis	0.000
2	Ko04712	Circadian rhythm—plant	0.000
3	Ko00196	Photosynthesis—antenna proteins	0.000
4	Ko04075	Plant hormone signal transduction	0.000
5	Ko00360	Phenylalanine metabolism	0.000
6	Ko00941	Flavonoid biosynthesis	0.001
7	Ko00904	Diterpenoid biosynthesis	0.001
8	Ko00940	Phenylpropanoid biosynthesis	0.004
9	Ko00906	Carotenoid biosynthesis	0.008
10	Ko00710	Carbon fixation in photosynthetic organisms	0.014
11	Ko00905	Brassinosteroid biosynthesis	0.043
12	Ko00350	Tyrosine metabolism	0.044
13	Ko02010	ABC transporters	0.046

### Analysis and verification of pathway mechanisms

**Figure 5:**
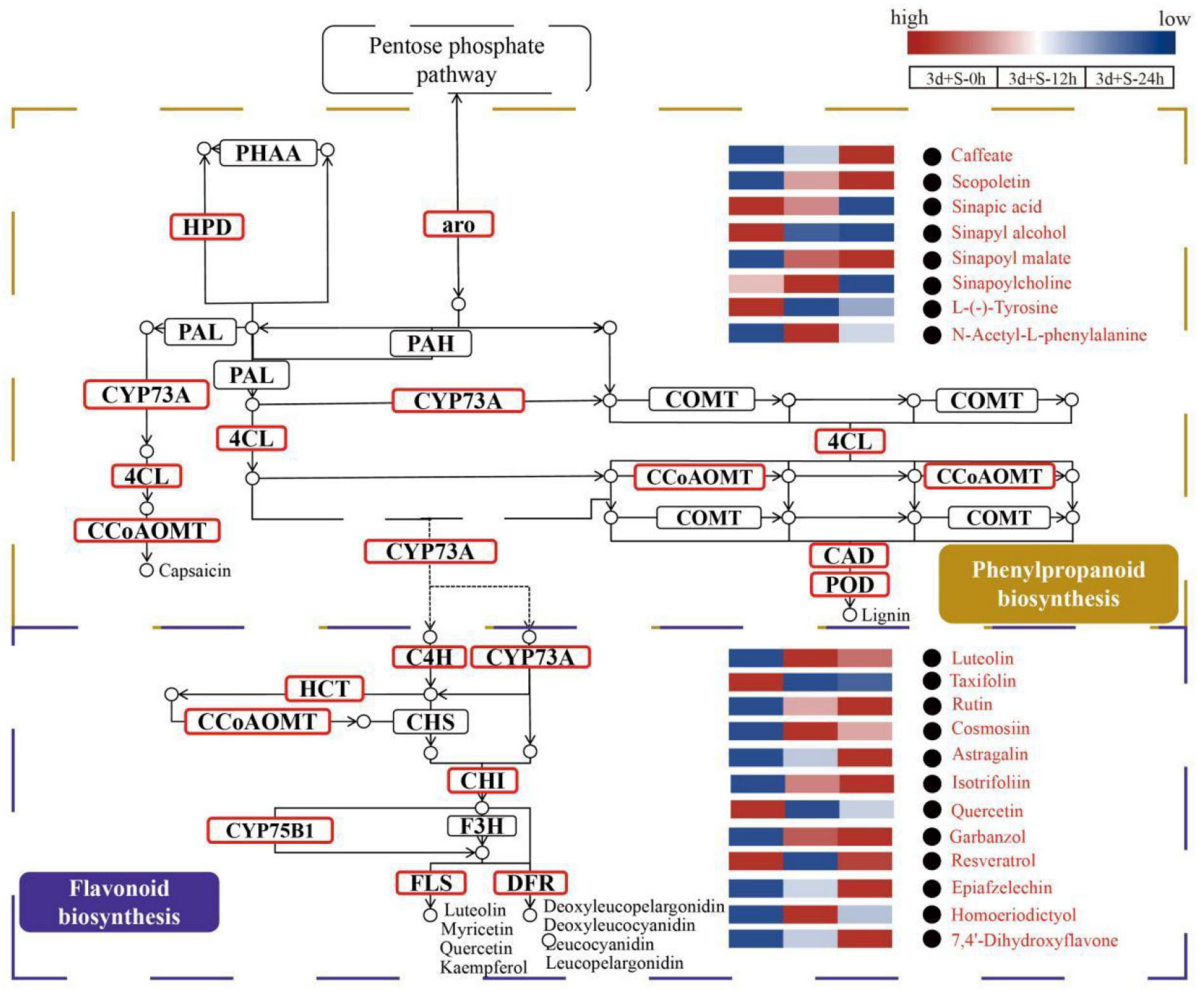
The enriched pathways analyzed by metabolomics and transcriptomics. The brown box represents the phenylpropanoid biosynthesis pathway, while the purple box represents the flavonoid biosynthesis pathway. The modules with red borders are differentially expressed genes (DEGs) enriched modules, while the red words with black circles are differentially altered metabolites (DAMs). Blue to red color represents expression from low to high.

The mechanisms of pathways enriched under salt stress are presented in Figure 5. Phenylpropanoid biosynthesis was the upstream pathway in which the metabolites, such as caffeate, scopoletin, sinapic acid, and sinapyl alcohol, were differentially expressed and directly affected the downstream pathway (flavonoid biosynthesis). Also, DEGs were enriched in some enzymes, including 4-coumarate-CoA ligase (4CL), caffeoyl-CoA o-methyltransferase, CAD, and POD. The flavonoid biosynthetic pathway was also enriched as the downstream pathway of phenylpropanoid biosynthesis, and the DEGs were enriched in some enzymes, such as FLS, CHI, and dihydroflavonol-4-reductase. A heatmap of DAMs (such as rutin, cosmosiin, luteolin, garbanzol, and quercetin) showed a significant change in the flavonoid biosynthesis pathway. Analysis of these two pathways revealed that the phenylpropanoid and flavonoid biosynthesis pathways could be crucial in salt stress response by promoting flavonoid accumulation.

**Figure 6:**
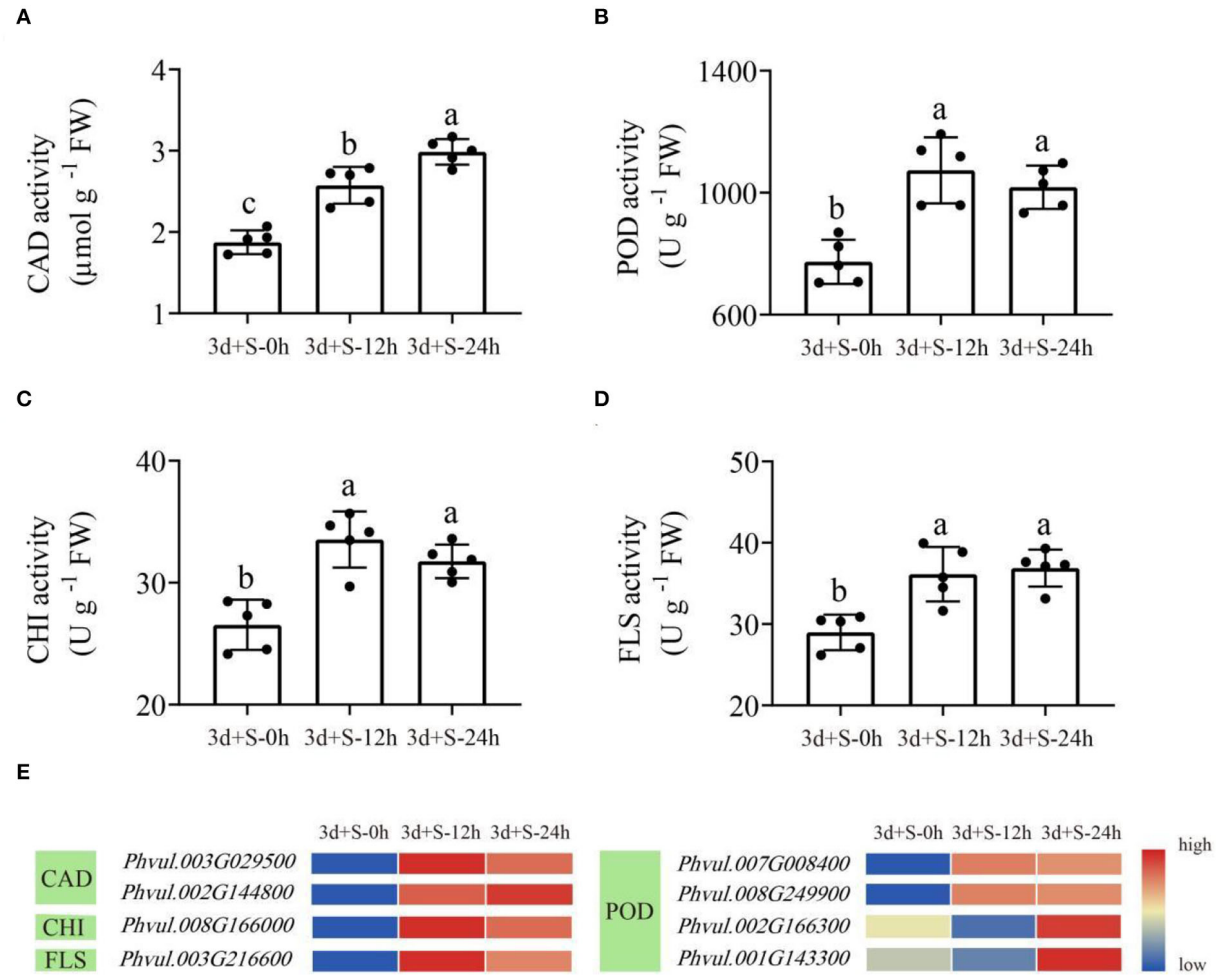
The enzyme activities in the enriched pathways at three time points. Lowercase letter(s) indicate significant differences (α = 0.05). Each black point represents the average value of replicates. **(A)** CAD activity; **(B)** POD activity; **(C)** CHI activity; **(D)** FLS activity; **(E)** the heatmap of differentially expressed genes (DEGs) enriched in these four enzymes. Blue to red color represents low to high expression.

**Figure 7:**
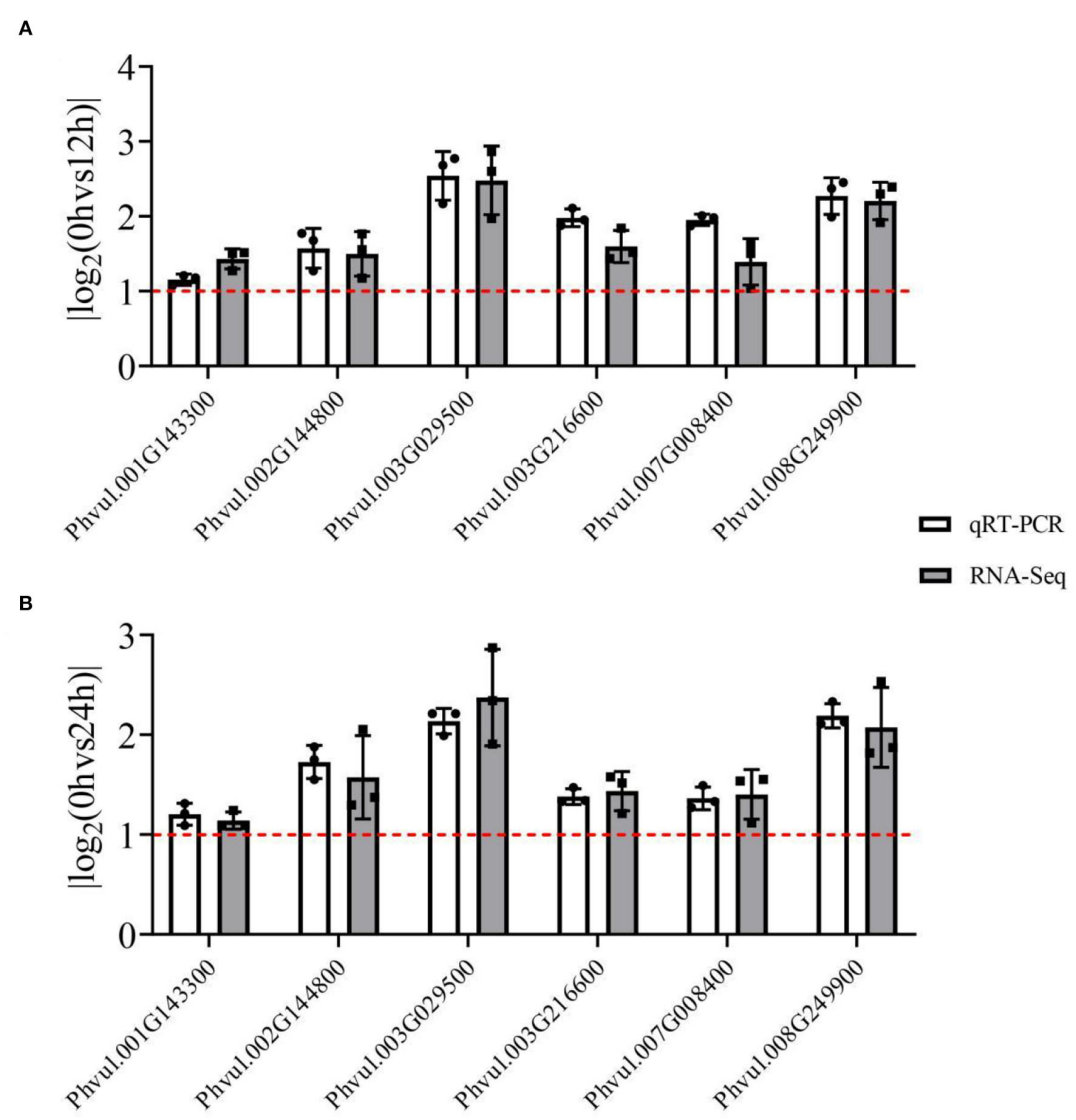
The qRT-PCR analysis of RNA-seq results of six differentially expressed genes (DEGs) from enriched pathways. The white grids represent the qRT-PCR results, while the gray grids represent the RNA-seq results. The red line represents the value of the |log2(FoldChange)|; a value >1 indicates that the genes were DEGs. Each black point represents the average value of replicates. **(A)** The |log2(FoldChange)| value of 3d+S-0h vs. 3d+S-12h; **(B)** the |log2(FoldChange)| value of 3d+S-0h vs. 3d+S-24h.

Herein, enzyme activity analysis in DEG-enriched points and qRT-PCR analysis was also done. Four enzyme activities that were DEG-enriched were tested. The results showed that salt stress significantly changed the activities of CAD, POD, CHI, and FLS (Figure 6). Besides, the DEGs heatmap showed that DEGs' expression associated with the enzymes differed significantly following salt exposure. Also, six different DEGs in these two pathways, which were enriched in CAD (*Phvul.002G144800* and *Phvul.003G029500*), POD (*Phvul.001G143300, Phvul.007G008400* and *Phvul.008G249900*), and FLS (*Phvul.003G216600*), were selected for qRT-PCR analysis while their expression and functional analysis are shown in Supplementary Table 9. The results showed that the values of |log_2_(FoldChange)| expression were all >1 (Figure 7), suggesting that these six genes had a significant expression change at the transcriptional level. Metabolome and transcriptome results revealed that phenylpropanoid and flavonoid biosynthesis responded to salt stress by promoting flavonoid accumulation.

## Discussion

### Flavonoid trends in germinating common beans

Seed germination is the beginning of most plant life cycles. When seeds (including legumes) begin to germinate, they break dormancy and restore physiological function, which induces metabolism and produces secondary metabolites and other nutrients (37). Rutin is often used as a flavonoid standard in botanical research (26, 38–40) because of its high content in legumes (41, 42). Similarly, the metabolome analysis conducted in this study revealed that germinating common beans have high levels of rutin. Here, the rutin content in germinating common beans initially increased, which illustrates that the beans were activated from hibernation and produced flavonoids (43). The rutin content then decreased after 96 h, which might be related to tissue differentiation after plant germination (44). Other nutrients in germinating beans also show similar trends; for instance, the γ-aminobutyric acid (GABA) content in germinating adzuki beans (*Vigna angularis*) showed increasing and decreasing, of which the highest content was at 48 h (37). The polyphenol content in germinating mung beans (*V. radiata*) also showed similar trends, and day 3 was the best time because it had the highest polyphenol content (45). Similarly, Lu and Guo (46) demonstrated that the vitamin C content of beans was at the highest level on day 3 and also showed an increasing and decreasing trend. Flavonoids (represented by rutin in this study) are important intermediates in plants' physiological metabolism, and their content also changes significantly during seed germination (47). Although secondary metabolites show these trends, the time elapsed for the levels to peak varies among species. This phenomenon might be because different nutrient types have different accumulation mechanisms, while different genotypes, or even different legume species, could also affect optimal times. In this study, rutin levels peaked at 96 h, which could be used as the optimal germination time.

### Short-term salt stress promotes rutin accumulation

Abiotic stress could influence grain quality and composition in crops (48) and significantly affect the flavonoid content in plants because flavonoids are an important regulator of plant responses to abiotic stress (49). As common abiotic stress, salt stress is important and accelerates the accumulation of certain plant metabolites (50, 51). Several studies have demonstrated that 70 mmol/L NaCl is the appropriate salt concentration for inducing salinity stress in the common bean (22, 52). Rutin is an important class of secondary metabolites widely found in plants and contributes to plant growth and development (16). Previous studies demonstrated that rutin content increases under stress, especially abiotic stress (42, 52, 53). Numerous studies found that flavonoids, including rutin, significantly accumulate under abiotic stress in many plant species, including *Arabidopsis*, maize (54), tomato (55), and green tea (56). However, flavonoids, including rutin, are stressful substances, and prolonged stress does not necessarily enable continued flavonoid accumulation (57). The antioxidant levels in soybean during drought showed an alternating increasing and decreasing trend, suggesting that short-term drought could rapidly enhance antioxidant intensities, while long-term droughts might cause plants to gradually adapt to these stresses and stop enhancement (58). Also, plant antioxidant enzyme activities showed a similar trend under abiotic stress, which revealed that short-term stress could increase plants' secondary metabolite production, while secondary metabolites under long-term stress might return to a lower level (59). In contrast, stress might inhibit plant germination and reduce flavonoid accumulation. The salt-tolerant common bean germinated better than the salt-sensitive variety, while oxidase enzyme activities of salt-sensitive variety produced insignificant differences at the sprouting stage, limiting plant growth and vitality (52). Here, short-term salt stress was more conducive to rutin accumulation in germinating common beans, and 3d+S could be used to study the mechanism of rutin accumulation under salt stress.

### The mechanism of flavonoid (rutin) accumulation under salt stress

In this study, phenylpropanoid and flavonoid biosynthesis were the two pathways that responded to salt stress and promoted flavonoid accumulation. Interestingly, phenylpropanoid biosynthesis was studied by a botanist researching the effects of abiotic stress on plant growth (60). Furthermore, phenylpropanoid biosynthesis is the pathway by which most flavonoids are produced, while enzyme activities in the phenylpropanoid biosynthetic pathway could alter the flavonoid accumulation rate (16). A key enzyme in phenylpropanoid metabolism is 4CL, which responds to abiotic stress, and the *4CL* genes have a function in regulating resilience (61). CAD is the final enzyme-catalyzed step in the phenylpropanoid biosynthesis pathway, and CAD activity is affected by salt stress, thus affecting the flavonoid accumulation rate (62). POD is a large family of plant-specific oxidoreductases, and POD activity can change significantly under salt stress (63).

**Figure 8:**
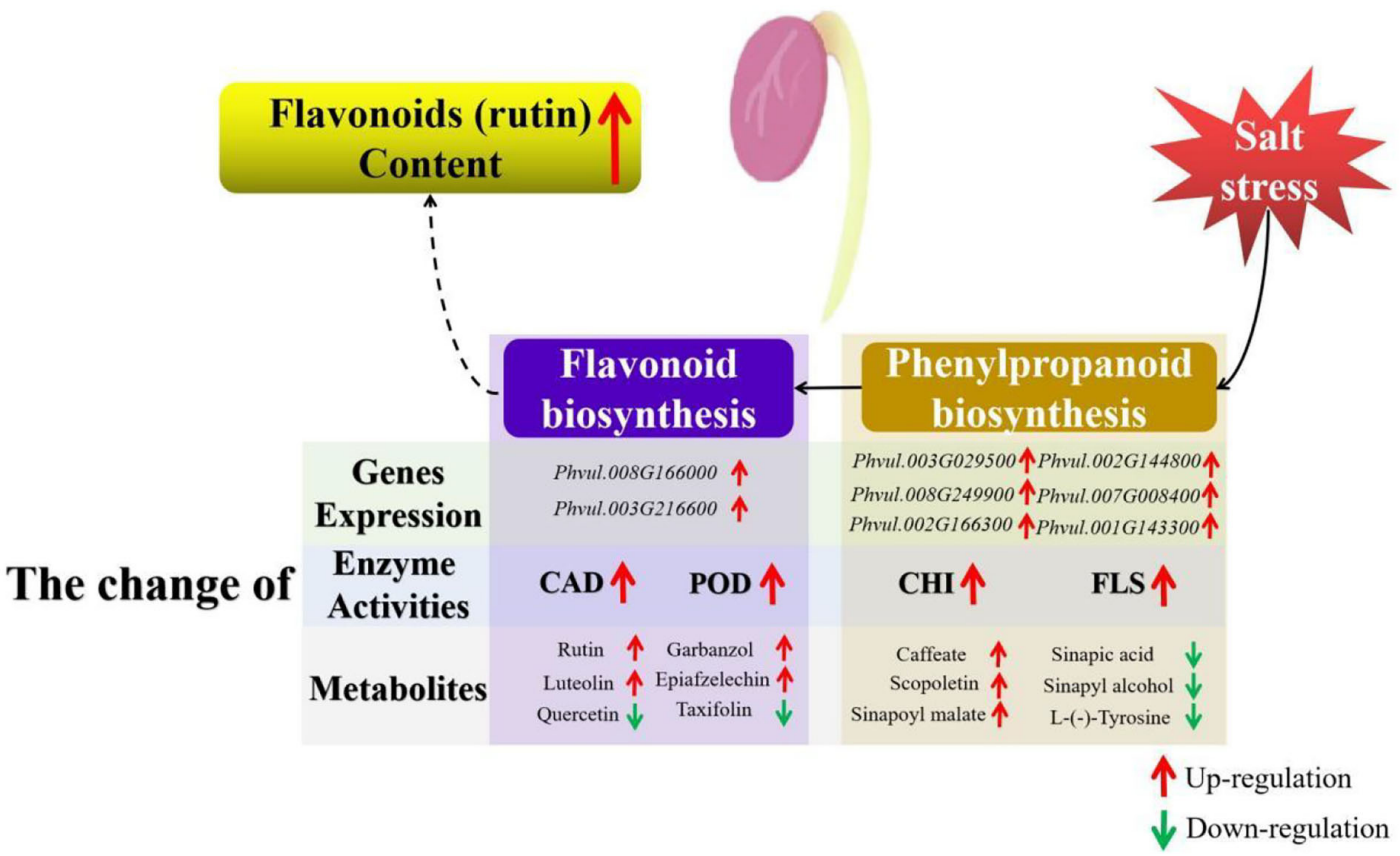
Schematic illustration of the mechanism of the flavonoid accumulation in germinating common beans under salt stress. The brown box represents the phenylpropanoid biosynthesis pathway, while the purple box represents the flavonoid biosynthesis pathway. Red arrows represent upregulation, while green arrows represent downregulation.

As the downstream pathway of phenylpropanoid biosynthesis, flavonoid biosynthesis is an important pathway for flavonoid accumulation (16). Some enzymes in the flavonoid metabolic pathway, such as CHI and FLS, have also been implicated in flavonoid accumulation and abiotic stress response (64). CHI catalyzes the conversion of bicyclic chalcone into tricyclic (2S)-flavanone and is linked to salt stress (65). FLS mediates the oxidation of dihydroflavonol to flavonol, while *FLS* gene upregulated and increased flavonoid content under salt stress (66). In this study, enzyme activities and expression levels were determined. The results revealed that salt stress affected the expression level of genes in the phenylpropanoid and flavonoid biosynthesis pathways and influenced the activities of CAD, POD, CHI, and FLS, which might be linked to flavonoid accumulation (Figure 8).

## Conclusion

In this study, rutin accumulation trends in germinating common beans were researched, and 96 h was the most suitable germination time because the sprouts had the highest rutin content. Sprouts (including epicotyls and hypocotyls) had more rutin content than cotyledons, hence they could be used as a target research tissue. Short-term salt treatment was more favorable for flavonoid accumulation than long-term salt treatment. Furthermore, metabolome and transcriptome analysis during short-term salt treatment revealed that phenylpropanoid and flavonoid biosynthesis are the enriched pathways that respond to salt stress and promote flavonoid accumulation. This study provides a rationale for germinating common beans and gives a new insight into the molecular mechanism of flavonoid accumulation in common beans under salt stress.

## Data availability statement

The transcriptome raw data was deposited to NCBI database, accession number PRJNA746732. The metabolome raw data was deposited to Zenodo database: doi.org/10.5281/zenodo.6820497.

## Author contributions

QZ: data curation and writing original draft. HS, GZ, JixZ, and ZZ: data curation. WZ, QW, and JinZ: conceptualization and methodology. JX and YG: software. Y-lD: formal data analysis and preparation of materials. QZ, ZY, and Y-lD: methodology and revised the manuscript. J-dD: data curation and funding acquisition. All authors contributed to the article and approved the submitted version.

## Funding

This study was financially supported by the National Key Research and Development Program (2020YFD1001402), the Research Project of Heilongjiang Bayi Agricultural University (XDB2011-02), and Special Funds from the Central Finance to Support the Development of Local Universities. The funding organizations had no role in experimental design, data collection, analysis, and interpretation of data or writing of the manuscript.

## Conflict of interest

The authors declare that the research was conducted in the absence of any commercial or financial relationships that could be construed as a potential conflict of interest.

## Publisher's note

All claims expressed in this article are solely those of the authors and do not necessarily represent those of their affiliated organizations, or those of the publisher, the editors and the reviewers. Any product that may be evaluated in this article, or claim that may be made by its manufacturer, is not guaranteed or endorsed by the publisher.

## Supplementary material

Supplementary Figure 1 The standard reference rutin curve.

Supplementary Figure 2 The proof of metabolome test.

Supplementary Table 1 Detailed information on the different treatments.

Supplementary Table 2 The qRT-PCR primers for differentially expressed genes (DEGs).

Supplementary Table 3 The detailed information of the metabolome analysis in this study.

Supplementary Table 4 The detailed information of differentially altered metabolites (DAMs) in the comparison of 3d+S-0h vs. 3d+S-24h.

Supplementary Table 5 The detailed information of differentially altered metabolites (DAMs) in the comparison of 3d+S-0h vs. 3d+S-12h.

Supplementary Table 6 The order of each compound expression in the five flavonoid kinds analyzed by metabolomics of differentially altered metabolites (DAMs) in the comparison of 3d+S-0h vs. 3d+S-12h or 3d+S-0h vs. 3d+S-24h.

Supplementary Table 7 The quality of the transcriptome results.

Supplementary Table 8 Transcriptome information uploaded to the NCBI database.

Supplementary Table 9 The expression and functional analysis of differentially expressed genes (DEGs) in qRT-PCR analysis.
