# Supplementary figures and images for: Molecular mechanisms of flavonoid accumulation in germinating common bean (Phaseolus vulgaris) under salt stress
Source: Front Nutr. 2022 Aug 29;9:928805. doi: 10.3389/fnut.2022.928805 (PMC9465018; doi:10.3389/fnut.2022.928805)

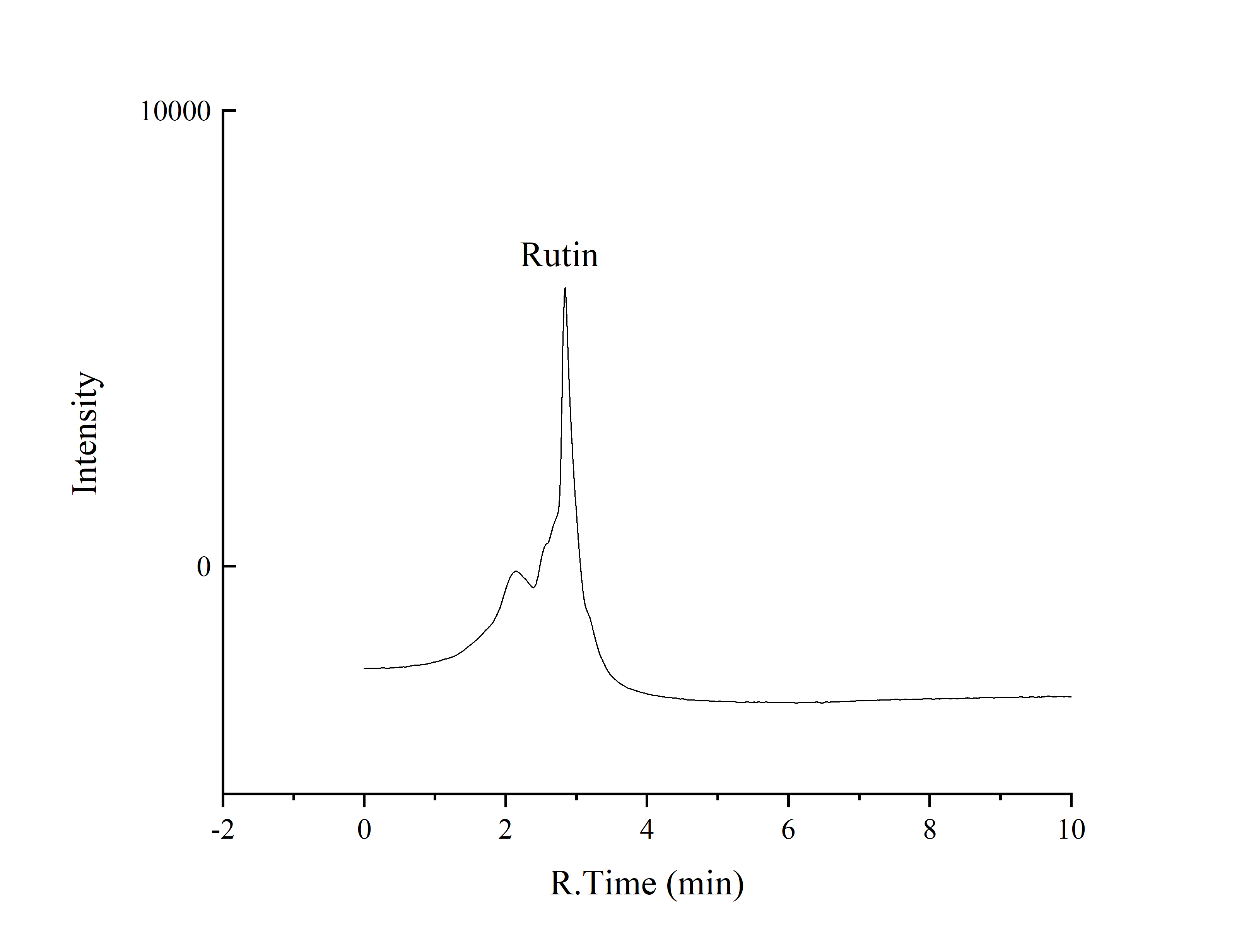

Supplement: Supplementary Figure 1 — The standard reference rutin curve. [file Data_Sheet_1.ZIP › Supplementary Figure/Figure S1.JPEG]

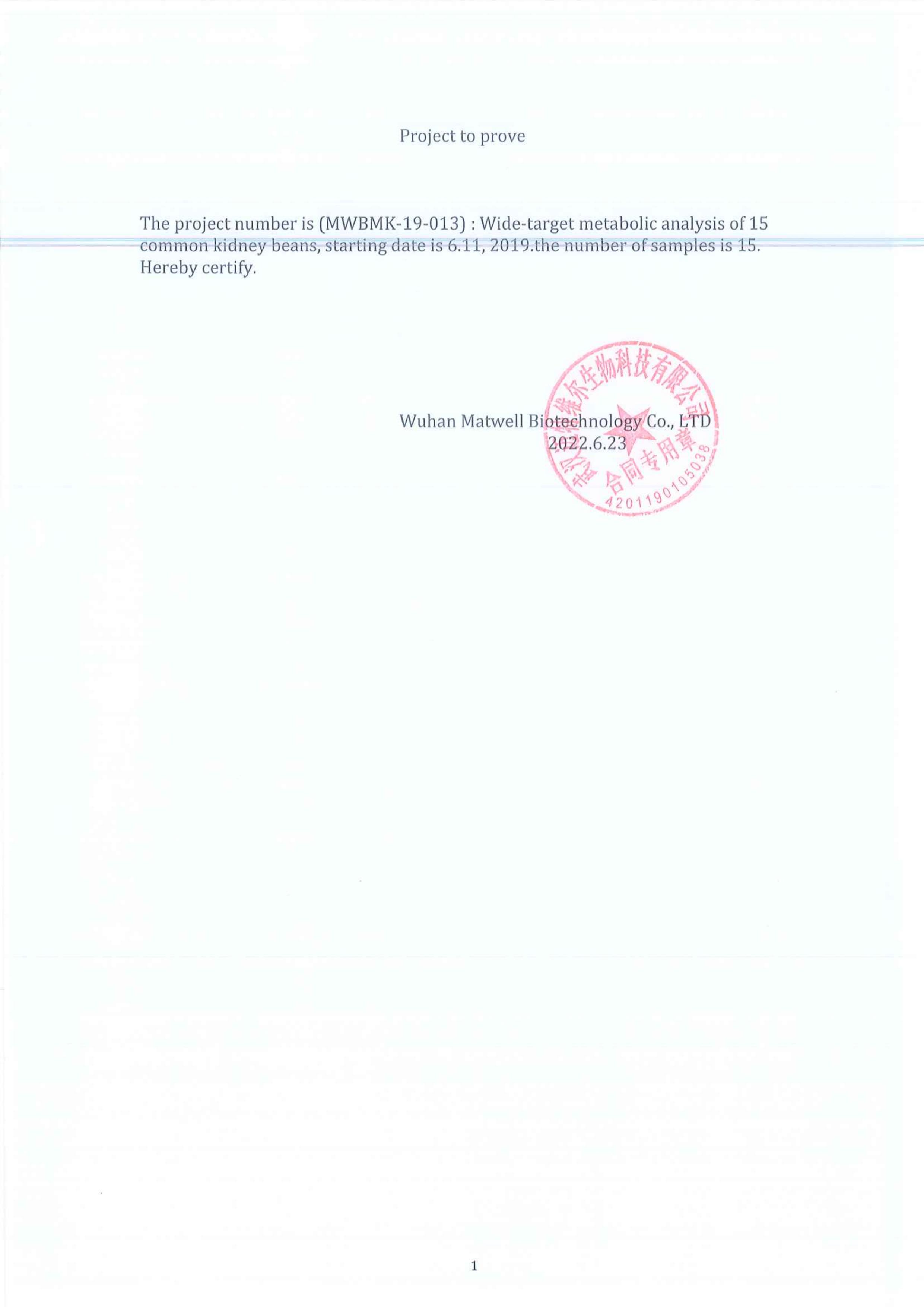

Supplement: Supplementary Figure 1 — The standard reference rutin curve. [file Data_Sheet_1.ZIP › Supplementary Figure/Figure S2.jpg]
