# Supplementary material for: Molecular mechanisms of flavonoid accumulation in germinating common bean (Phaseolus vulgaris) under salt stress
Source: Front Nutr. 2022 Aug 29;9:928805. doi: 10.3389/fnut.2022.928805 (PMC9465018; doi:10.3389/fnut.2022.928805)
Supplement: Supplementary Table 1 — Detailed information on the different treatments. [file Data_Sheet_2.ZIP › supplyment table/Table S1.docx]

**Table S1:** Detailed information of the different treatments*.*

| No. | Name of treatments | Detailed information in different treatments |
| --- | --- | --- |
| 1 | 0d+S | The treatment adding salt at the 0 day. |
| 2 | 1d+S | The treatment adding salt at the 1 day. |
| 3 | 2d+S | The treatment adding salt at the 2 day. |
| 4 | 3d+S | The treatment adding salt at the 3 day. |
| 5 | 4d | The treatment without salt stress for 4 days. |
