# Supplementary material for: Molecular mechanisms of flavonoid accumulation in germinating common bean (Phaseolus vulgaris) under salt stress
Source: Front Nutr. 2022 Aug 29;9:928805. doi: 10.3389/fnut.2022.928805 (PMC9465018; doi:10.3389/fnut.2022.928805)
Supplement: Supplementary Table 1 — Detailed information on the different treatments. [file Data_Sheet_2.ZIP › supplyment table/Table S2.docx]

**Table S2:** The qRT-PCR primers for differentially expressed genes (DEGs).

| Primer | Sequence (5’→3’) |
| --- | --- |
| ***Ref-Act11***-F | TGCATACGTTGGTGATGAGG |
| ***Ref-Act11***-R | AGCCTTGGGGTTAAGAGGAG |
| ***Phvul.001G143300-***F | CTGTGTCTTTGGGAGGAGG |
| ***Phvul.001G143300-***R | TAATGCAACGAGATCGGTT |
| ***Phvul.002G148800-***F | ATATACCAGCTTGCTCTTC |
| ***Phvul.002G148800-***R | TTTCAACTTTACTTCTCCC |
| ***Phvul.003G029500-***F | CTCCCCATTTTTGTTCTCC |
| ***Phvul.003G029500***-R | CTTTGCTTCCCACCTCTGT |
| ***Phvul.003G216600***-F | ACACCTCCTTCTTCTACCA |
| ***Phvul.003G216600***-R | TTCTCTTCCAAATAAACCC |
| ***Phvul.007G008400***-F | TTTCCAAATGCTAACTCCCT |
| ***Phvul.007G008400***-R | TTGAATCTCTTCTGCCTAAT |
| ***Phvul.008G249900-***F | GACTGTTTTGTCAATGGATG |
| ***Phvul.008G249900-***R | TTACTTCAAAACCTCTCAAA |
