# Supplementary material for: Molecular mechanisms of flavonoid accumulation in germinating common bean (Phaseolus vulgaris) under salt stress
Source: Front Nutr. 2022 Aug 29;9:928805. doi: 10.3389/fnut.2022.928805 (PMC9465018; doi:10.3389/fnut.2022.928805)
Supplement: Supplementary Table 1 — Detailed information on the different treatments. [file Data_Sheet_2.ZIP › supplyment table/Table S7.docx]

Table S7: The quality of the transcriptome results.

| No.-ID | Total Reads | Clean reads | Mapped Reads | GC Content | %≥Q30 |
| --- | --- | --- | --- | --- | --- |
| 0h-1 | 61573266 | 30786633 | 84.43% | 45.21% | 93.31% |
| 0h-2 | 54652380 | 27326190 | 84.56% | 45.13% | 93.38% |
| 0h-3 | 64151544 | 32075772 | 84.17% | 45.35% | 93.18% |
| 12h-1 | 60991266 | 30495633 | 84.43% | 45.75% | 93.26% |
| 12h-2 | 63947458 | 31973729 | 84.18% | 45.44% | 93.12% |
| 12h-3 | 58312902 | 29156451 | 84.28% | 45.53% | 92.97% |
| 24h-1 | 58043820 | 29021910 | 84.65% | 45.46% | 93.27% |
| 24h-2 | 61228112 | 30614056 | 84.25% | 45.60% | 93.28% |
| 24h-3 | 63162164 | 31581082 | 84.02% | 45.52% | 92.92% |
