# Supplementary material for: Molecular mechanisms of flavonoid accumulation in germinating common bean (Phaseolus vulgaris) under salt stress
Source: Front Nutr. 2022 Aug 29;9:928805. doi: 10.3389/fnut.2022.928805 (PMC9465018; doi:10.3389/fnut.2022.928805)
Supplement: Supplementary Table 1 — Detailed information on the different treatments. [file Data_Sheet_2.ZIP › supplyment table/Table S8.docx]

Table S8: Transcriptome information uploaded to the NCBI database.

| Title | Accession | Study | Bioproject  accession | Biosample  accession | Design  description | Filename | |
| --- | --- | --- | --- | --- | --- | --- | --- |
| CK1 | SRR15166902 | SRP328568 | PRJNA746732 | SAMN20239299 | Water treatment | CK1good_1.fq | CK1good_2.fq |
| CK2 | SRR15166901 | SRP328568 | PRJNA746732 | SAMN20239300 | Water treatment for repeated | CK2good_1.fq | CK2good_2.fq |
| CK3 | SRR15166900 | SRP328568 | PRJNA746732 | SAMN20239301 | Water treatment for repeated again | CK3good_1.fq | CK3good_2.fq |
| 12h1 | SRR15166899 | SRP328568 | PRJNA746732 | SAMN20239302 | 12h salt treatment | 12H1_good_1.fq | 12H1_good_2.fq |
| 12h2 | SRR15166898 | SRP328568 | PRJNA746732 | SAMN20239303 | 12h salt treatment for repeated | 12H2_good_1.fq | 12H2_good_2.fq |
| 12h3 | SRR15166897 | SRP328568 | PRJNA746732 | SAMN20239304 | 12h salt treatment for repeated again | 12H3_good_1.fq | 12H3_good_2.fq |
| 24h1 | SRR15166896 | SRP328568 | PRJNA746732 | SAMN20239305 | 24h salt treatment | 24H1_good_1.fq | 24H1_good_2.fq |
| 24h2 | SRR15166895 | SRP328568 | PRJNA746732 | SAMN20239306 | 24h salt treatment for repeated | 24H2_good_1.fq | 24H2_good_2.fq |
| 24h3 | SRR15166894 | SRP328568 | PRJNA746732 | SAMN20239307 | 24h salt treatment for repeated again | 24H3_good_1.fq | 24H3_good_2.fq |
