# Supplementary material for: Molecular mechanisms of flavonoid accumulation in germinating common bean (Phaseolus vulgaris) under salt stress
Source: Front Nutr. 2022 Aug 29;9:928805. doi: 10.3389/fnut.2022.928805 (PMC9465018; doi:10.3389/fnut.2022.928805)
Supplement: Supplementary Table 1 — Detailed information on the different treatments. [file Data_Sheet_2.ZIP › supplyment table/Table S9.docx]

**Table S9:** The expression and functional analysis of differentially expressed genes (DEGs) in qRT-PCR analysis.

| Gene ID | Sigificant  3d+S-0h *vs* 3d+S-12h | Sigificant  3d+S-0h *vs* 3d+S-24h | Functions of genes |
| --- | --- | --- | --- |
| *Phvul.001G143300* | Down | Up | POD |
| *Phvul.002G144800* | Up | Up | CAD |
| *Phvul.003G029500* | Up | Up | CAD |
| *Phvul.003G216600* | Up | Up | FLS |
| *Phvul.007G008400* | Up | Up | POD |
| *Phvul.008G249900* | Up | Up | POD |
